# Effects of Autologous Blood‐Derived Extracellular Vesicles on Skin Regeneration and Anti‐Aging: A Clinical Study

**DOI:** 10.1111/jocd.70574

**Published:** 2025-12-30

**Authors:** Hwansu Kang, Ji‐Hwan Kim, Youin Cho, Dam Go, Jihyun Park, Hyung‐Gi Kim, Sanghoon Park

**Affiliations:** ^1^ Advanced Regenerative Medicine Center ID Hospital Seoul Korea; ^2^ Department of Plastic Surgery ID Hospital Seoul Korea; ^3^ Department of Chemistry University of Michigan Ann Arbor Michigan USA

**Keywords:** anti‐aging, autologous, clinical study, dermatology, extracellular vesicle, rejuvenation, skin

## Abstract

**Introduction:**

Autologous blood‐derived extracellular vesicles (EVs) have gained attention as a novel therapeutic approach for skin rejuvenation. This study evaluates their efficacy in improving wrinkles, lifting, hydration, barrier function, tone, radiance, texture, and pore size in human subjects.

**Aim:**

This study aimed to evaluate the clinical efficacy of a single intradermal injection of autologous blood‐derived EVs in improving multiple skin parameters, including wrinkle reduction, skin lifting, hydration, barrier function, tone, radiance, texture, and pore size, over a three‐week period.

**Methods:**

Twenty‐five participants aged 40–59 underwent a single facial injection of autologous blood‐derived EVs. Evaluations were conducted before treatment, 5 days post‐treatment, and 3 weeks post‐treatment using objective skin analysis tools and subjective self‐assessment questionnaires.

**Results:**

Statistically significant improvements (*p* < 0.05) were observed in wrinkle depth, skin lifting, hydration, elasticity, and barrier function at both 5‐day and 3‐week time points. Skin tone, radiance, texture, and pore size also improved significantly over the study period. Subjective self‐assessments corroborated the instrumental findings, with a marked increase in participant‐reported satisfaction. No adverse reactions or safety concerns were reported during the study.

**Conclusions:**

This study demonstrates the clinical efficacy and safety of autologous blood‐derived EVs in facial rejuvenation, supporting their potential as a non‐invasive cosmetic treatment.

## 1 Introduction

Skin aging is a multifaceted biological process influenced by both intrinsic and extrinsic factors. Intrinsic contributors, such as genetic predisposition and cellular senescence, drive natural aging processes, while extrinsic factors, including ultraviolet (UV) radiation, environmental factors, and lifestyle choices, further accelerate skin deterioration [1]. These influences collectively lead to a decline in skin integrity, characterized by decreased collagen and elastin, compromised barrier function, increased transepidermal water loss (TEWL), and the visible manifestation of wrinkles [2, 3]. Despite the availability of various cosmetic and medical treatments—such as topical retinoids, laser therapy, and dermal fillers—the demand persists for minimally invasive, biologically driven, and more effective skin rejuvenation strategies [4].

Advancements in regenerative medicine have recently underscored the therapeutic potential of extracellular vesicles (EVs), particularly exosomes, in dermatology [5]. EVs are nanoscale (30–150 nm) particles that facilitate intercellular communication by delivering bioactive molecules, including proteins, lipids, microRNAs (miRNAs), and cytokines [6, 7]. These vesicles are secreted by various cell types (e.g., keratinocytes, fibroblasts, mesenchymal stem cells (MSCs), and platelets) and play fundamental roles in tissue regeneration, immune modulation, and extracellular matrix (ECM) remodeling [8, 9].

Due to their ability to stimulate fibroblast proliferation, enhance collagen synthesis, mitigate oxidative stress, and promote wound healing, EV‐based therapies have emerged as a promising approach in dermatology [10]. Preclinical and clinical research has demonstrated that MSCs‐ and platelet‐derived EVs can drive dermal regeneration and attenuate visible signs of aging through paracrine signaling mechanisms [11, 12, 13]. However, concerns surrounding the immunogenicity, batch‐to‐batch variability, and complex regulatory landscape of allogeneic EV therapies have led researchers to explore autologous EV sources as a viable alternative [14].

Autologous blood‐derived EVs offer distinct advantages over traditional stem cell‐derived EVs. By utilizing a patient's own biological material, they eliminate the risk of immune rejection and pathogen transmission while maintaining high biocompatibility and therapeutic potential [15]. Although research on autologous blood‐derived EVs in dermatology remains limited, preliminary studies suggest notable benefits, including enhanced skin hydration, improved elasticity, and reduction in wrinkle formation [13]. Given their regenerative potential, further well‐structured clinical investigations are warranted to comprehensively evaluate their efficacy and safety profile.

Recent progress in dermatologic research has increasingly recognized EVs as promising mediators of skin regeneration and anti‐aging therapy. Advances demonstrate that EVs derived from mesenchymal stem cells, fibroblasts, and even plants can promote fibroblast proliferation, enhance collagen synthesis, and reduce oxidative stress in photo‐aged skin [16, 17, 18]. Contemporary reviews further highlight that exosome‐based interventions in cosmetic dermatology remain in early clinical phases but show significant potential for enhancing skin elasticity, hydration, and barrier repair [19, 20]. These findings underscore the biological rationale for evaluating autologous blood‐derived EV preparations in a clinical setting focused on skin rejuvenation.

The aim of this study is to assess the efficacy of a single intradermal injection of autologous blood‐derived EVs in enhancing skin regeneration over a three‐week period. Additionally, this study evaluates to establish a safety profile for the treatment, providing essential clinical evidence to support the integration of autologous EV‐based therapies into dermatological practice.

## 2 Materials and Methods

### 2.1 Study Design

This single‐center, prospective clinical study was conducted at an independent clinical research organization. The study protocol and informed‐consent documents were reviewed and approved by the Institutional Review Board (IRB No. GIRB‐24N07‐HG) in accordance with the ethical principles of the Declaration of Helsinki (1964) and its later amendments.

A total of 25 healthy adults (11 in their 40s and 14 in their 50s; 18 females and 7 males; mean ± SD = 49.6 ± 3.3 years) were enrolled. Inclusion criteria included adults aged 40–59 years presenting with mild‐to‐moderate facial photoaging and visible wrinkles, Fitzpatrick skin types II–IV, and willingness to refrain from other cosmetic procedures during the study period. Exclusion criteria comprised pregnancy or lactation, known allergies to study materials, active dermatologic or systemic disorders, anticoagulant use, a history of hypertrophic scarring or keloids, and participation in other clinical trials within the previous 3 months. Each participant received a single intradermal injection of autologous EV preparation, evenly distributed across the entire facial area at the mid‐to‐deep dermal layer using a 33G needle.

Clinical evaluations were conducted at baseline (Day 0), Day 5, and Week 3 under controlled environmental conditions (temperature 20°C–24°C, relative humidity 45%–55%) following a 30‐min acclimation period. All participants provided written informed consent before enrollment.

### 2.2 Autologous Blood Derived EVs Preparation

Autologous blood‐derived EVs were isolated from peripheral blood samples collected and processed on the same day. Whole blood was centrifuged at 3500 rpm for 10 min to obtain plasma, followed by a second centrifugation at 10 000 × *g* for 10 min to remove remaining cellular debris. The EV fraction was purified from 2 mL of plasma using size‐exclusion chromatography (SEC) with a 35 nm qEV1 column (Izon Science Ltd., New Zealand). Before plasma loading, the column was equilibrated with 27 mL of sterile normal saline to remove storage preservatives and stabilize the matrix.

The first 4.7 mL of eluent was discarded, and the subsequent 2.8 mL containing the EV‐enriched fraction was collected. Nanoparticle tracking analysis (NTA, NanoSight PRO, Malvern Panalytical, UK) was used to determine particle concentration and size distribution. The modal diameter of the recovered particles was approximately 90 nm, which falls within the size range (30–150 nm) typically reported for small EVs according to the Minimal Information for Studies of Extracellular Vesicles (MISEV) guidelines [21].

The SEC‐based isolation and NTA profiling confirmed the successful enrichment of small EVs while minimizing contamination by soluble plasma proteins, providing a standardized and reproducible source of autologous EVs suitable for intradermal application.

### 2.3 Assessment of Wrinkles, Skin Lifting, Texture, and Pore Parameters

All participants were acclimated for 30 min under controlled environmental conditions (20°C–24°C temperature and 45%–55% relative humidity) prior to measurement. Wrinkle depth, surface roughness, and pore morphology were analyzed using an Antera 3D CS system (Miravex Ltd., Dublin, Ireland), which employs multi‐directional LED illumination and multispectral 3D reconstruction to generate high‐resolution surface topography maps.

Wrinkle depth and wrinkle volume in the periorbital and nasolabial regions were determined using the wrinkle analysis mode, while overall lifting effects were quantified through the volumetric lifting index based on skin surface displacement. Surface texture was characterized by the arithmetic mean roughness value (Ra, expressed in micrometers), and pore morphology was quantified as total pore area (mm^2^) and mean pore volume (mm^3^). Each region was measured three times, and mean values were used for analysis. A reduction in wrinkle depth, Ra, and pore area together with an increase in the lifting index was interpreted as an improvement in skin surface smoothness and firmness.

### 2.4 Assessment of Skin Elasticity, Hydration, and Barrier Function

Skin biomechanical and barrier parameters were measured on the cheek region using standardized devices (Courage + Khazaka Electronic GmbH, Cologne, Germany) after a 30‐min acclimation under 20°C–24°C and 45%–55% RH conditions.

Skin elasticity was evaluated with a Cutometer Dual MPA 580 using a 2 mm probe and a negative pressure of 450 mbar. Each measurement included three suction–release cycles (1 s each), and the elasticity parameter R2 (Ur/Ue) was calculated automatically to represent total skin elasticity, with higher R2 values indicating greater elasticity. Skin hydration was assessed using a Corneometer CM 825, which measures the electrical capacitance of the stratum corneum, and the mean of three readings was expressed in arbitrary units (AU). Transepidermal water loss (TEWL) was measured with a Tewameter TM 300 using an open‐chamber probe, with each measurement lasting 25 s and the mean of the final 3 s expressed in g/m^2^ h. All measurements were performed in duplicate on both cheeks, and averaged values were used for analysis.

### 2.5 Assessment of Skin Tone and Radiance

Skin tone and radiance were analyzed using a Visia‐CR imaging system (Canfield Scientific Inc., Fairfield, NJ, USA) equipped with a Canon EOS 5D digital camera and a six‐filter illumination wheel. Standardized photographs were acquired under two predefined imaging modes to ensure reproducibility and comparability across time points.

In the Standard2 mode, overall skin brightness was quantified by calculating the *L*‐value (lightness) within the CIELAB color space, which objectively represents skin luminance. In the Parallel‐polarized mode, surface radiance intensity was determined based on polarized light reflection analysis, providing an index of skin luminosity and gloss. All image data were processed using I‐Max plus software (ING PLUS Co., Korea) under identical illumination and camera positioning at each time point (baseline, Day 5, and Week 3 post‐treatment). Increases in *L*‐value and radiance intensity were interpreted as enhanced skin tone and overall brightness.

### 2.6 Safety and Adverse Event Assessment

Safety was assessed in all participants based on adverse events (AEs) identified during the study period. All AEs that occurred after treatment, including any reactions related to the test procedure or spontaneously during follow‐up, were documented. At each visit (baseline, 5 days, and 3 weeks after treatment), a dermatologist evaluated the injection sites and recorded any local reactions such as erythema, edema, tenderness, induration, or pruritus. Participants were also asked about any systemic symptoms that appeared during the observation period. The overall incidence rate of AEs was calculated using all reported events, and the results were used as reference data for the product's safety evaluation. No significant or serious AEs were reported, and no participant discontinued the study because of treatment‐related reactions. Therefore, the autologous blood‐derived EV preparation was considered safe for clinical use during this study.

### 2.7 Measurement of Skin Density

Skin density was assessed using a Skin Scanner (TPM, Germany). The instrument uses a 22 MHz ultrasound transducer that generates short electrical pulses to create a density image and extract quantitative data. Both epidermal and dermal density values (%) were analyzed, and an increase in these combined density values (epidermis + dermis) indicates an improvement in skin compactness. Measurements were performed at three time points: before treatment, 5 days after treatment, and 3 weeks after treatment. All scans were conducted under controlled temperature (20°C–24°C) and humidity (45%–55%) conditions after a 30‐min acclimation period, as specified in the study protocol.

### 2.8 Subjective Self‐Assessment Evaluation

To evaluate participant‐reported outcomes following the administration of autologous blood‐derived extracellular vesicle (EV) treatment, a structured self‐assessment questionnaire was conducted in accordance with the approved clinical protocol (GMEA‐MT‐24074).

A total of 25 participants completed the survey at two post‐treatment time points—Day 5 and Week 3 after treatment. The questionnaire comprised 11 items designed to assess perceived improvements in wrinkle appearance, skin firmness, hydration, barrier function, skin tone, radiance, texture, pore refinement, elasticity, and overall skin condition. Each item was rated on a five‐point Likert scale, ranging from “Strongly agree (5)” to “Strongly disagree (1)”, as defined in the clinical report. Responses of 3 points or higher (“Neutral,” “Agree,” or “Strongly Agree”) were categorized as positive responses, and the percentage of positive ratings was calculated for each parameter at both assessment time points. Participants completed the questionnaire independently under standardized conditions to minimize response bias. Any treatment‐related discomfort or adverse effects mentioned in the self‐assessment form were reviewed and verified by the investigator during the same visit. For presentation purposes, five representative parameters—wrinkle, elasticity, hydration, tone, and pore appearance—were selected to visualize the overall trend of subjective improvement in the Results section.

### 2.9 Statistical Analysis

All statistical analyses were conducted using IBM SPSS Statistics, version 27.0 (IBM Corp., USA). Prior to inferential testing, the Shapiro–Wilk test was used to assess the normality of the data distribution. For datasets that satisfied the normality assumption, repeated‐measures analysis of variance (ANOVA) was applied to compare values across the three time points (baseline, 5 days, and 3 weeks after treatment). The sphericity assumption was verified by Mauchly's test, and when the assumption was not met, Wilks' lambda from the multivariate test was interpreted. Differences among the time points were examined using within‐subject contrast analysis. If the normality assumption was not satisfied, the non‐parametric Friedman test was used instead. Pairwise comparisons were then performed using the Wilcoxon signed‐rank test, and the significance level was adjusted using the Bonferroni correction for multiple comparisons.

All analyses adopted a two‐sided significance threshold of *p* < 0.05. Statistical significance is denoted as **p < 0.05 when analyzed by repeated‐measures ANOVA and ^*#*^ *p* < 0.05 when analyzed by the Wilcoxon signed‐rank test, consistent with the original clinical report.

## 3 Result

### 3.1 Reduction in Wrinkle Depth and Improvement in Skin Firmness

**FIGURE 1:**
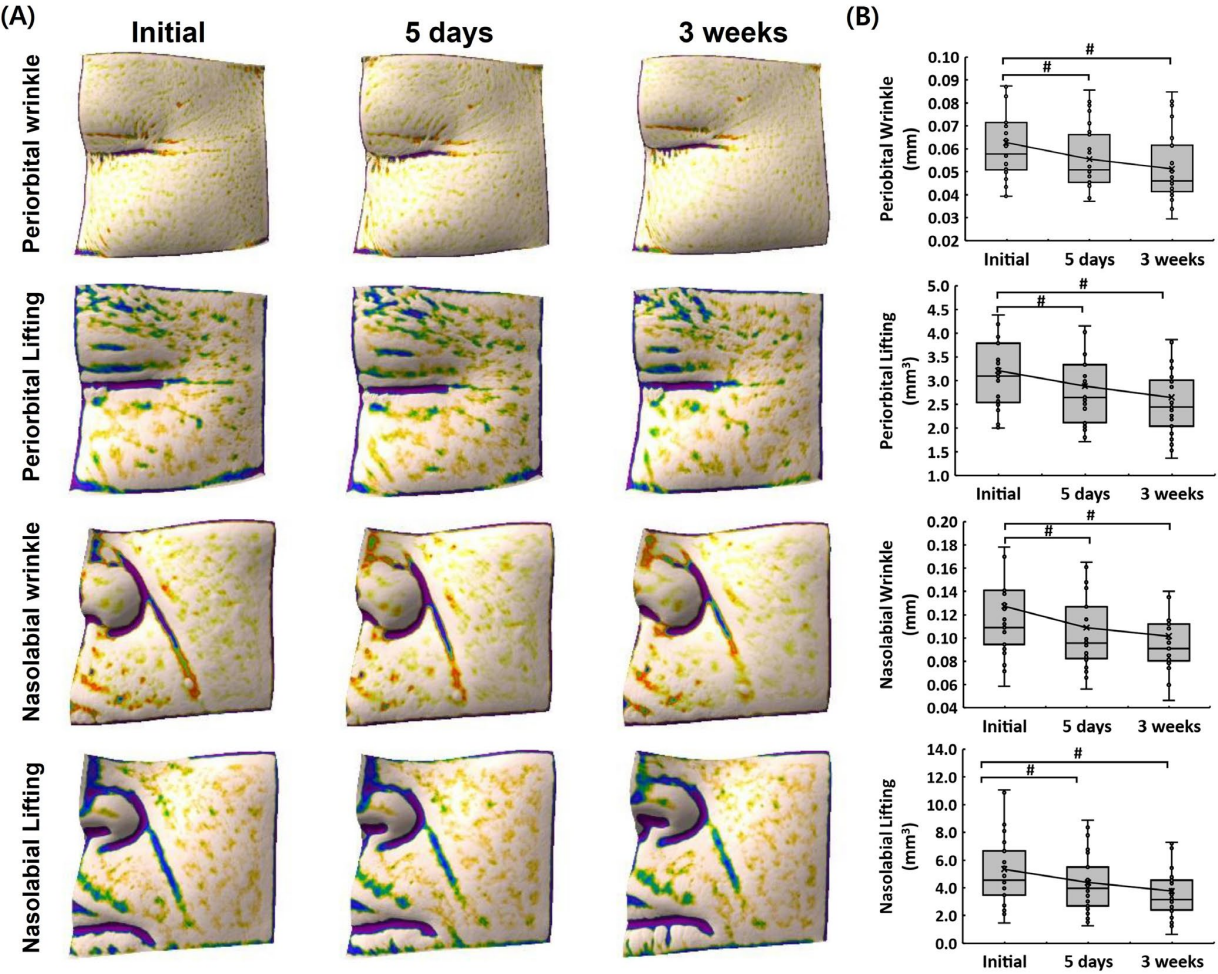
Three‐dimensional (3D) imaging and quantitative analysis of periorbital and nasolabial wrinkle depth and lifting effects before treatment, at 5 days, and at 3 weeks post‐treatment. (A) Representative 3D images showing periorbital wrinkles and lifting, as well as nasolabial wrinkles and lifting at different time points. (B) Quantitative analysis of periorbital wrinkles and lifting, along with nasolabial wrinkles and lifting, based on data from 25 clinical trial participants. Data are expressed as mean ± standard deviation. Statistical significance is indicated by *#p* < 0.05, analyzed using the Wilcoxon signed‐rank test.

Three‐dimensional imaging demonstrated a reduction in periorbital and nasolabial wrinkles along with improvements in skin lifting effects over time. As shown in Figure 1A, periorbital wrinkles visibly decreased following treatment. Quantitative analysis confirmed a 19.05% reduction in periorbital wrinkle depth after 3 weeks (Figure 1B). Similarly, nasolabial wrinkles showed a 19.68% decrease compared to baseline (Figure 1B). The lifting effect was also evident in both the periorbital and nasolabial areas. As depicted in Figure 1A, a noticeable enhancement in skin firmness was observed post‐treatment. Corresponding analysis showed a 17.55% reduction in periorbital lift volume and a 29.38% decrease in nasolabial lift volume at 3 weeks (Figure 1B), indicating improved structural support and skin elasticity.

### 3.2 Enhancement of Skin Hydration, Barrier Function, Radiance, and Elasticity

**FIGURE 2:**
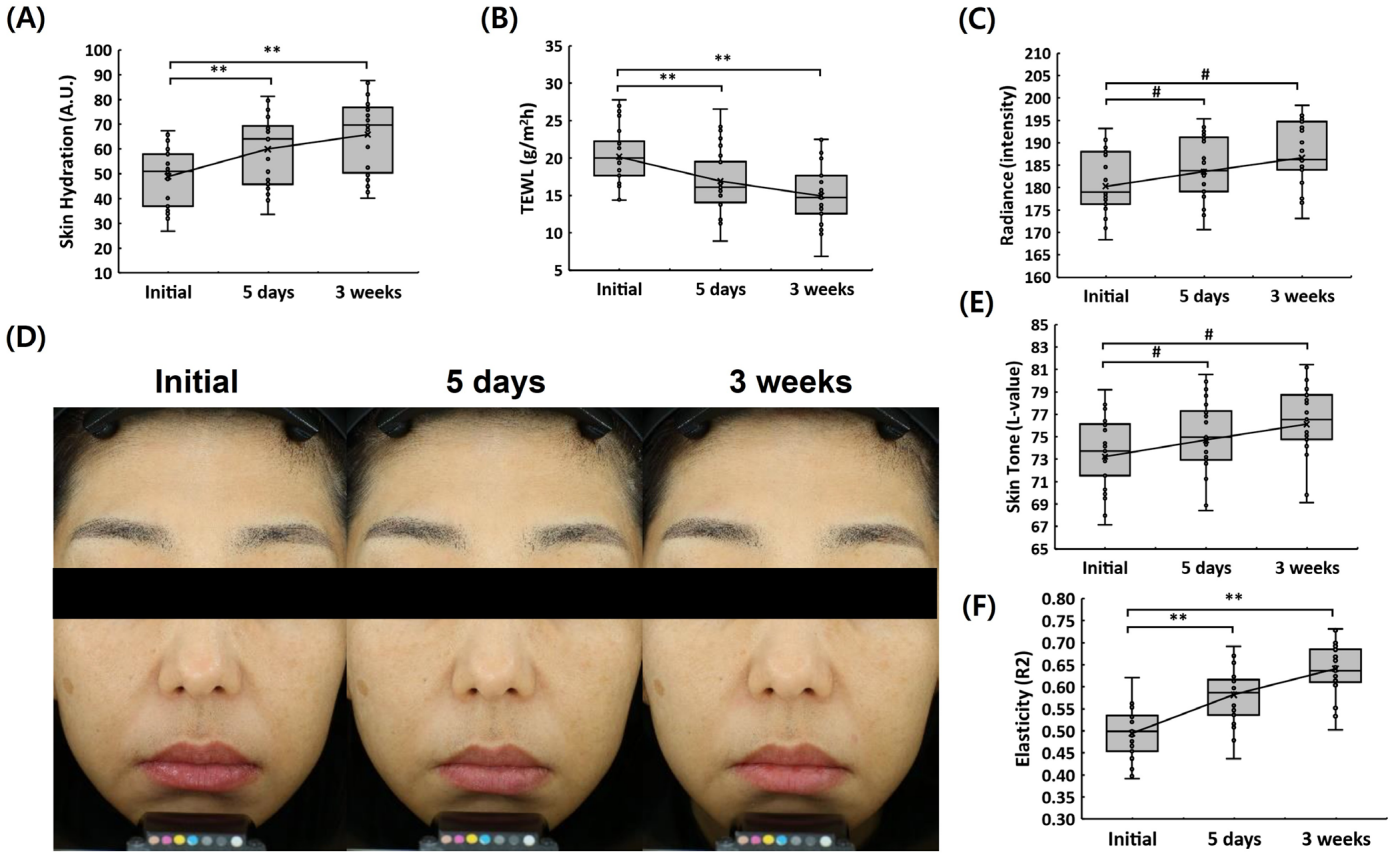
Skin clinical evaluation results from a total of 25 participants before treatment, at 5 days, and at 3 weeks post‐treatment. (A) Graph showing changes in skin hydration over time. (B) Trans‐epidermal water loss (TEWL) measurements indicating improvements in skin barrier function. (C) Quantitative analysis of skin radiance intensity. (D, E) Representative facial images and corresponding graph demonstrating changes in skin tone (*L*‐value). (F) Graph showing the analysis of skin elasticity (R2) at different time points. Data are presented as mean ± standard deviation. Statistical significance is indicated as **p < 0.05 by repeated measures ANOVA and *#p* < 0.05 by Wilcoxon signed‐rank test.

The evaluation of skin hydration demonstrated a significant increase following EV treatment. As shown in Figure 2A, hydration levels improved by 22.65% at 5 days and 34.52% at 3 weeks compared to baseline. Additionally, TEWL analysis indicated a 16.17% reduction at 5 days and a 25.85% reduction at 3 weeks, suggesting an enhancement in skin barrier function (Figure 2B). These findings indicate that EV treatment effectively improves both skin hydration and barrier integrity.

Assessment of skin radiance and tone further confirmed the benefits of treatment. As depicted in Figure 2C, radiance intensity increased significantly over time, reflecting a brighter complexion. Similarly, Figure 2D illustrates an enhancement in skin tone, with significant improvements observed at 5 days and 3 weeks post‐treatment. These changes suggest that EV application contributes to a visibly healthier and more radiant appearance.

Skin elasticity, measured using the R2 parameter, also exhibited a marked improvement. As presented in Figure 2E, elasticity increased by 17.81% at 5 days and 29.76% at 3 weeks, indicating a progressive enhancement in skin firmness and resilience.

### 3.3 Enhancement of Skin Texture, Pore Reduction, and Dermal Density

**FIGURE 3:**
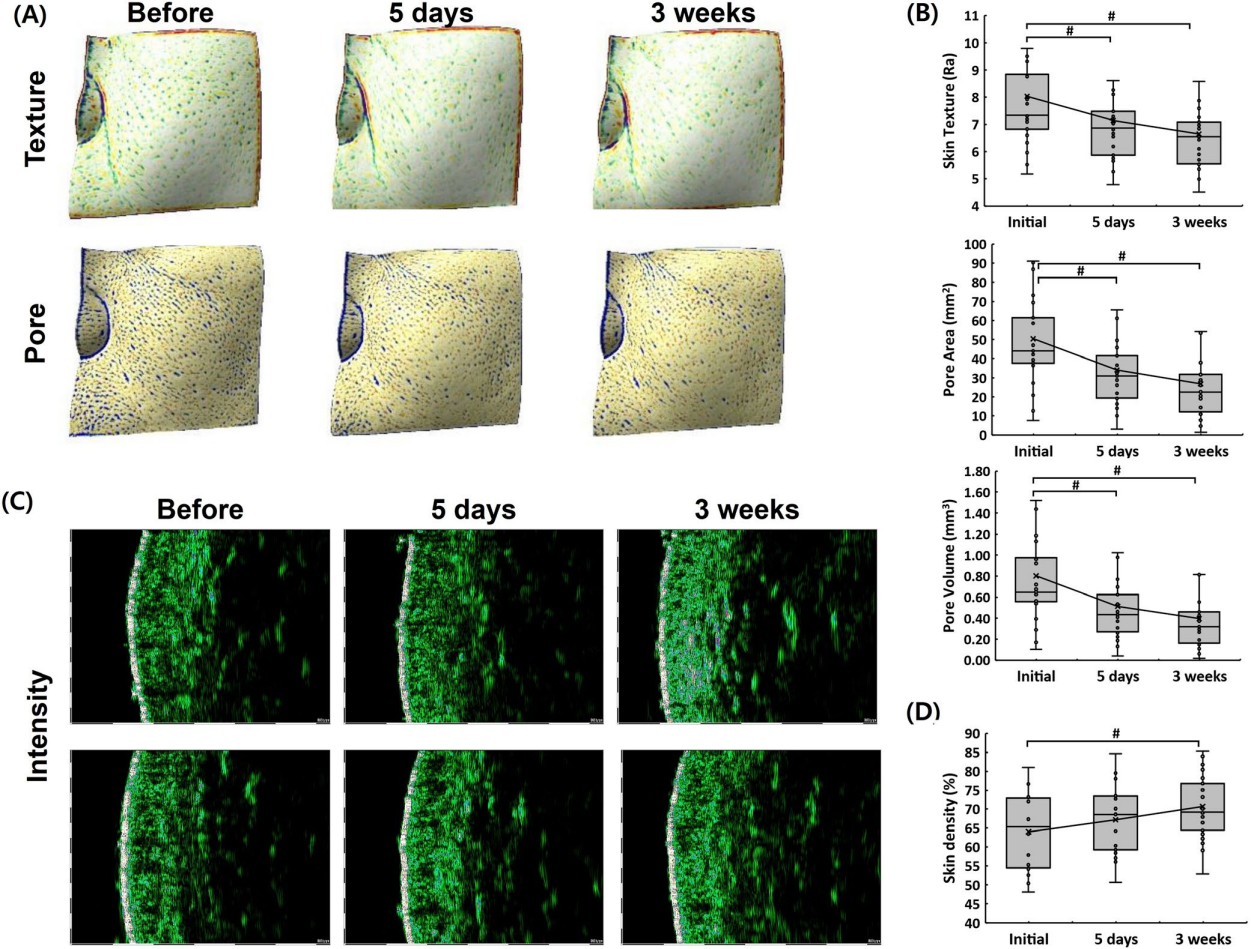
Three‐dimensional imaging and quantitative assessment of skin texture, pores, and skin density over time. (A) Representative 3D images showing changes in skin texture and pore structure before treatment, at 5 days, and at 3 weeks post‐treatment. (B) Quantitative analysis of skin texture roughness (Ra), pore area, and pore volume in 25 clinical trial participants, demonstrating progressive improvements over time. (C) Ultrasound‐based cross‐sectional images representing changes in skin structure and density before and after treatment. (D) Quantitative box plot analysis of skin density (%) measured from clinical trial participants, indicating an increase in density over time. Data are expressed as mean ± standard deviation. Statistical significance is indicated by *#p* < 0.05, analyzed using the Wilcoxon signed‐rank test.

Three‐dimensional imaging demonstrated a progressive enhancement in skin texture and pore refinement following EV treatment. As illustrated in Figure 3A, the skin surface became noticeably smoother over time, with a reduction in roughness and improved uniformity. Quantitative analysis revealed a 17.11% decrease in skin roughness (Ra) at 3 weeks post‐treatment (Figure 3B), confirming the significant improvement in skin texture.

In addition to texture refinement, pore characteristics were also evaluated. The results indicated a substantial decline in pore size and volume, with a 32.35% reduction in pore area at 5 days, which further decreased to 46.80% at 3 weeks. Similarly, pore volume showed a 36.16% decrease at 5 days and a 50.62% reduction at 3 weeks (Figure 3B). These findings suggest that EV treatment effectively minimizes pore size and contributes to a more refined skin appearance.

To further investigate changes in skin structure, ultrasound‐based imaging was used to assess skin density. As depicted in Figure 3C, post‐treatment cross‐sectional images revealed increased dermal density. Quantitative analysis confirmed a 10.43% increase in skin density at 3 weeks (Figure 3D), suggesting that the treatment promotes improved dermal integrity and structural reinforcement.

### 3.4 Sustained Skin Improvement Based on Subjective Self‐Assessment

**FIGURE 4:**
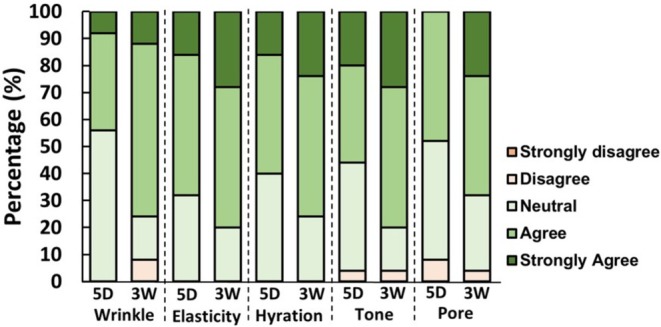
Stacked bar graphs illustrating the distribution of participant responses in the subjective self‐assessment of wrinkle reduction, skin elasticity, hydration, skin tone, and pore refinement at 5 days (5D) and 3 weeks (3W) post‐treatment. Responses were evaluated using a five‐point Likert scale (Strongly Disagree, Disagree, Neutral, Agree, Strongly Agree). A progressive increase in the proportion of positive responses (Agree and Strongly Agree) was observed over time, suggesting sustained improvements in perceived skin parameters following treatment.

The participant‐reported outcomes, evaluated through a structured self‐assessment survey, indicated a progressive improvement in multiple skin parameters following the administration of autologous blood‐derived EVs. As depicted in Figure 4, responses regarding wrinkle reduction, skin elasticity, hydration, skin tone, and pore refinement demonstrated an upward trend in positive perception from 5 days post‐treatment to 3 weeks post‐treatment, suggesting sustained treatment efficacy.

A notable increase in the proportion of participants selecting “Agree” or “Strongly Agree” was observed across all parameters at 3 weeks post‐treatment. Wrinkle reduction was perceived as significantly improved over time, with a higher percentage of participants reporting favorable responses at 3 weeks compared to 5 days post‐treatment. Similarly, skin elasticity showed a progressive increase in positive responses, suggesting improvements in dermal firmness and structural integrity. Hydration levels and barrier function were also subjectively rated higher at 3 weeks, reflecting enhanced moisture retention and epidermal protection. Improvements in skin tone and pore appearance followed a comparable trend, with participants perceiving a more uniform complexion and refined skin texture over time. In addition to these key parameters, subjective evaluations of overall skin radiance, texture smoothness, and general satisfaction with treatment outcomes also exhibited a consistent increase (data not shown). These findings collectively suggest that a single session of autologous blood‐derived EV therapy provides both an immediate and progressively sustained improvement in skin quality, with effects persisting for at least 3 weeks post‐treatment.

## 4 Discussion

This study investigated the clinical effects of autologous blood‐derived EV therapy on skin regeneration, focusing on wrinkle reduction, skin firmness, hydration, barrier function, radiance, elasticity, texture, pore size, and skin density over a three‐week period. The findings demonstrated that a single intradermal injection of autologous EVs led to statistically significant improvements across all assessed parameters, highlighting the potential of this treatment as an effective, minimally invasive approach for skin rejuvenation.

The isolation protocol employed in this study adheres to the methodological framework recommended by the MISEV 2023 consensus [21], combining SEC‐based purification with nanoparticle tracking analysis to verify the recovery of nanosized EVs. This standardized approach ensured the consistent acquisition of EV‐enriched plasma fractions within the expected biophysical range and minimized interference from non‐vesicular plasma components. Given the clinical design of this investigation, this operational definition of EVs provides a reliable foundation for assessing their regenerative efficacy while maintaining translational relevance for aesthetic dermatology practice. These findings indicate that EV‐based therapy could represent a novel and biologically compatible approach for improving overall skin quality and dermal regeneration.

The clinical improvements observed—particularly in elasticity, pore reduction, and texture—align with recent evidence suggesting multi‐pathway mechanisms for EV treatments. According to recent meta‐analyses and systematic reviews, EVs can modulate extracellular matrix remodeling, attenuate MMP activity, and enhance angiogenesis in dermal tissues [9, 22, 23, 24]. Moreover, the evolving landscape of aesthetic EV therapy emphasizes both opportunities and challenges, such as standardization of EV isolation and delivery, which reinforces the need for methodologically rigorous clinical studies like the present investigation [25, 26].

The reduction in periorbital and nasolabial wrinkles observed in this study is consistent with previous research suggesting that EV‐based therapies enhance collagen synthesis and fibroblast activity, leading to visible improvements in skin elasticity and wrinkle depth [8, 27]. The 19.05% decrease in periorbital wrinkle depth and 19.68% reduction in nasolabial wrinkles at 3 weeks post‐treatment suggest that EV therapy effectively counteracts age‐related collagen degradation and ECM deterioration. Additionally, the observed decrease in periorbital and nasolabial lift volumes by 17.55% and 29.38%, respectively, indicates an improvement in dermal structural integrity and skin firmness. These outcomes align with prior studies showing that EVs promote fibroblast proliferation, collagen deposition, and elastin production, thereby supporting tissue remodeling and skin tightening. The regenerative properties of EVs have been attributed to their cargo, which includes miRNAs, growth factors, and cytokines known to influence ECM turnover, reduce oxidative stress, and modulate inflammatory pathways [28]. Given the growing demand for non‐invasive and biologically driven anti‐aging interventions, these results reinforce the potential of EV‐based treatments in dermatology.

Beyond wrinkle reduction and lifting effects, improvements in skin hydration and barrier function were also evident. The 34.52% increase in skin hydration and 25.85% reduction in TEWL suggest that EV therapy enhances moisture retention and strengthens epidermal integrity. These effects may be linked to the presence of exosomal bioactive molecules, such as hyaluronic acid‐binding proteins and lipid metabolism regulators, which have been shown to contribute to epidermal barrier reinforcement [29]. Additionally, increased radiance and improved skin tone post‐treatment indicate that EV therapy may help counteract pigmentation irregularities and oxidative stress‐induced pigmentation [30]. The observed enhancement in skin elasticity, with a 29.76% increase at 3 weeks, further supports the hypothesis that EV therapy plays a role in dermal remodeling by restoring fibroblast function and promoting the synthesis of structural proteins. These findings are consistent with previous studies demonstrating the ability of EVs to enhance fibroblast activity and improve overall skin resilience [30, 31].

EV therapy also demonstrated a significant impact on skin texture, pore size, and skin density. The 17.11% reduction in skin roughness and 50.62% decrease in pore volume indicate that the treatment effectively refines skin texture and minimizes pore visibility [32]. These improvements may be attributed to the anti‐inflammatory and remodeling properties of exosomal cargo, which promote keratinocyte turnover and regulate sebum production, leading to a smoother and more uniform skin surface [33]. Furthermore, the 10.43% increase in skin density, as confirmed by ultrasound‐based imaging, suggests that EV therapy strengthens both the epidermal and dermal layers, contributing to overall structural enhancement. Given that skin density is a crucial factor in maintaining youthful and resilient skin, these findings highlight the broader regenerative potential of autologous EVs in dermatological applications.

Collectively, the results of this study provide encouraging evidence supporting the efficacy of autologous blood‐derived EV therapy in skin regeneration. The observed improvements across multiple skin parameters highlight its potential as a biologically compatible, minimally invasive approach for skin rejuvenation [34], although larger controlled studies with extended follow‐up are warranted to confirm these findings and refine treatment protocols. Future research should also elucidate the molecular mechanisms underlying EV‐mediated skin regeneration to further enhance the therapeutic precision of this emerging modality.

## 5 Conclusions

These findings support the therapeutic potential of autologous blood‐derived EV therapy as a minimally invasive and biologically driven approach for skin rejuvenation. By utilizing autologous biological material, this therapy reduces the risk of immune rejection and pathogen transmission, offering advantages over traditional allogeneic or synthetic alternatives. However, while this study provides encouraging evidence supporting its efficacy, additional research with larger sample sizes, extended follow‐up periods, and mechanistic investigations is warranted to further validate the long‐term benefits and optimize treatment protocols. Future studies should also focus on elucidating the precise molecular pathways through which EVs mediate skin regeneration, enabling more targeted and effective therapeutic applications in dermatology.

## Author Contributions

H.K. and J.‐H.K. designed the research study. S.P. provided resources, H.K. drafted the original paper, and J.P., H.‐G.K., and S.P. reviewed and edited the paper. J.‐H.K., Y.C. and D.G. performed the research.

## Ethics Statement

The study protocol received approval from the Institutional Review Board of Global Medical Research Center (GIRB‐24N07‐HG).

## Consent

Written informed consent was obtained from all participants, including specific consent for the use of clinical photographs for publication and presentation.

## Conflicts of Interest

The authors declare no conflicts of interest.

## Data Availability

The data that support the findings of this study are available on request from the corresponding author. The data are not publicly available due to privacy or ethical restrictions.
